# A case of linear atrophoderma of Moulin^☆^^☆☆^

**DOI:** 10.1016/j.abd.2019.03.005

**Published:** 2019-12-18

**Authors:** Li-Wen Zhang, Meng-Sha Ma, Tao Chen, Li-Xin Fu

**Affiliations:** Department of Dermatovenereology, Chengdu Second People's Hospital, Sichuan, China

Dear Editor,

A 15-year-old Chinese girl presented with a 10-year history of asymptomatic, unilateral light brown patches affecting the right arm and right side of the trunk. The lesions were asymptomatic. There were no prior skin lesions or inflammation. There was no significant medical or family history. Physical examination found linear hyperpigmented atrophic patches on the right arm and right trunk following Blaschko's lines, involving both the anterior and posterior aspects. The skin was slightly atrophic on palpation. No signs of induration or inflammation were noted (Fig. 1A and B). Laboratory investigations – including full blood count, erythrocyte sedimentation rate, liver function test, renal profile, and antinuclear antibodies – were all negative or within the normal range. Biopsy of a lesion showed a normal epidermis with increased pigmentation of the basal layer, with more compact dermal collagen and mild upper dermal perivascular lymphocytic infiltration (Fig. 2). Dermoscopy found multiple light brown networks with unclear margins. The patient was diagnosed with linear atrophoderma of Moulin (LAM) and started treatment with topical halometasone 0.5% cream and hydroquinone 2% cream for two months, with no improvement.

**Figure 1:**
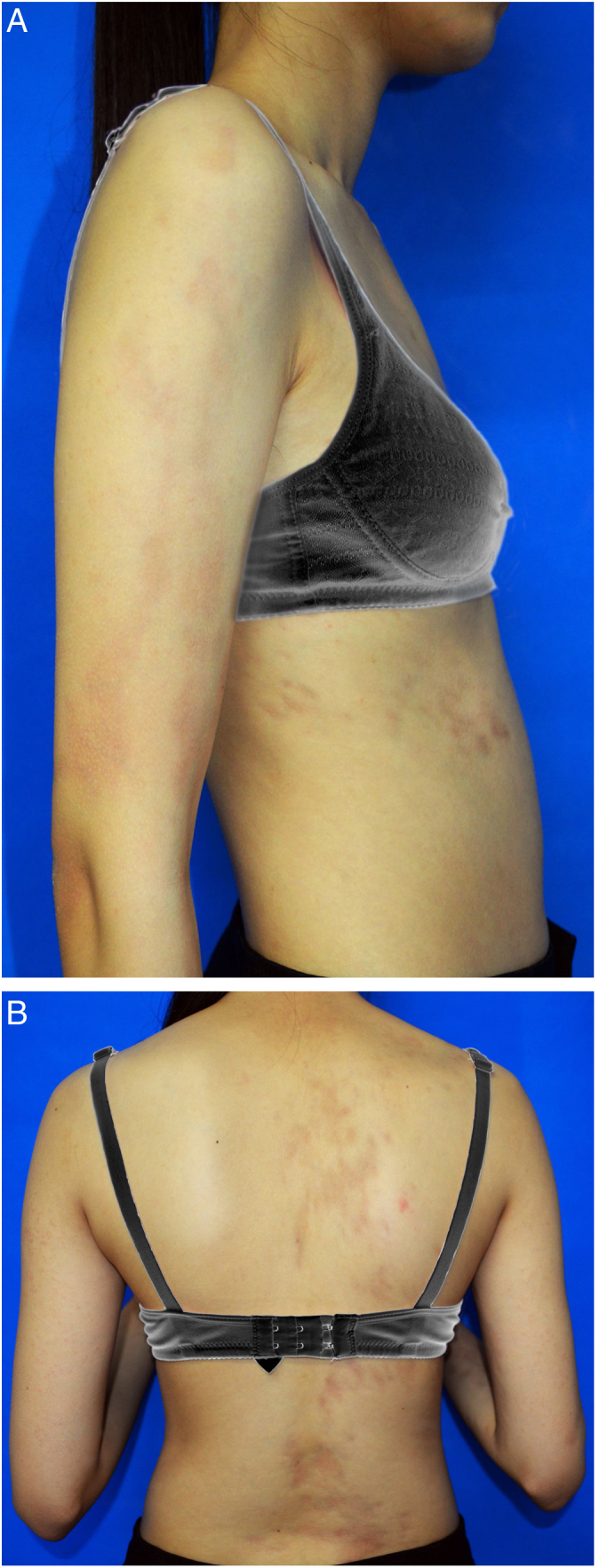
(A) Linear hyperpigmented patches on the right arm and right trunk following Blaschko's lines. (B) Linear hyperpigmented patches on the right arm and right trunk following Blaschko's lines.

**Figure 2:**
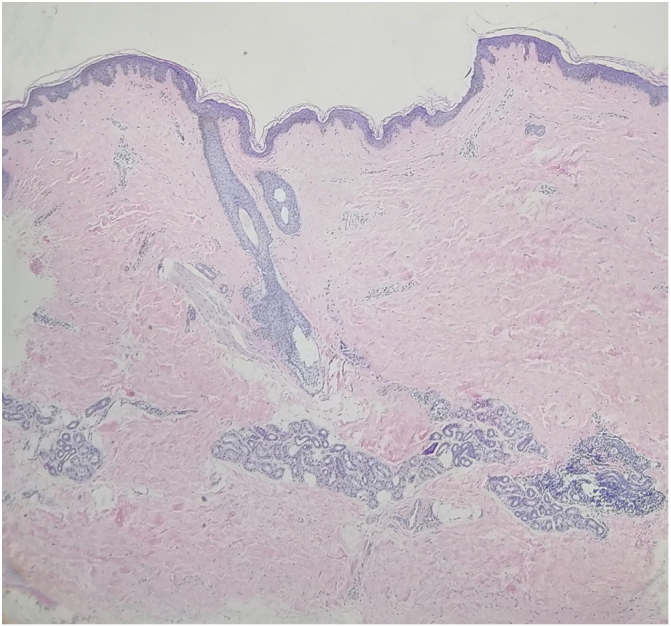
A normal epidermis with increased pigmentation of the basal layer, with more compact dermal collagen and mild upper dermal perivascular lymphocytic infiltration (Hematoxylin & eosin, ×40).

LAM is a rare and distinct clinical entity characterized by acquired unilateral, hyperpigmented, and atrophic bandlike skin lesions following the lines of Blaschko, without prior inflammation or sclerotic appearance. It is named after Moulin, who, in 1992, reported on five patients with pigmented and more-or-less atrophic bands along Blaschko's lines.^1^ LAM usually progresses as a linear atrophic lesion in the first few months; then the lesion ceases to progress and persists. The etiology of LAM remains unclear. All reported cases were so far sporadic. It may be connected with gene mosaicism or autoimmunity. A study of the atrophic component of LAM by ultrasonography revealed that subcutaneous volume reduction was the cause of the atrophic appearance, not dermal atrophy.^2^ Even though the clinical manifestation of LAM is rather unique, the histopathology of LAM is quite inconspicuous. Hematoxylin and eosin staining usually shows hyperpigmentation only in basal epidermal layers, without abnormal collagen or elastic fibers in the dermis or any obvious inflammation.^1^ There may be some perivascular lymphocytic infiltration, acanthosis, epidermal atrophy, altered collagen in the dermis, and decreased or fragmented elastic tissue.^2^

Lopez et al.^3^ proposed the following diagnostic criteria for -LAM, including: (1) Onset during childhood or adolescence; (2) Development of hyperpigmented, slightly atrophic, unilateral lesions following Blaschko lines on the trunk or limbs; (3) Absence of prior inflammation or subsequent scleroderma; (4) A stable, non-progressive clinical course without a pattern of remission; (5) Histologic findings showing hyperpigmentation of the basal epidermis and a normal dermis with unaltered connective tissue and elastic fibers. Up to now, more than 30 cases of LAM have been reported in the literature. However, the condition may be overestimated. If the diagnostic criteria are strictly adhered to, the diagnosis of LAM cannot be reached in some cases, as these authors reported histologic findings that are compatible with other clinical entities.^3^

LAM must be differentiated from atrophoderma of Pasini and Pierini (APP), which also presents with similar configuration, atrophy, and hyperpigmentation, but does not follow Blaschko's lines. In addition, LAM is different from linear morphea, which usually presents preceding inflammation, induration, or scleroderma.

Histopathologically, morphea shows collagen bundles that are closely packed and oriented horizontally, and dermal appendages and subcutaneous fat are progressively lost. However, it is still debated whether LAM is a distinct entity. There are many clinical and histologic similarities between LAM, APP, and morphea, thus some of the literature suggests that these diseases represent part of a disease spectrum, and that LAM may not be a distinct entity.^4^ LAM may be a Blaschko-linear variant of APP, and APP may be considered to be an abortive form of morphea.^4^

There is no effective treatment for LAM. Topical corticosteroids and heparin were not helpful. Some trial treatments showed partial response to LAM, including the following: topical calcipotriol, systemic methotrexate or aminobenzoate, and intralesional platelet-rich plasma therapy.^5^ The current report presented a case of LAM with classic clinical and histopathological features.

## Financial support

None declared.

## Authors’ contribution

Li-Wen Zhang and Meng-Sha Ma contributed equally to this work. Li-Wen Zhang: Approval of the final version of the manuscript; conception and planning of the study; composition of the manuscript; critical review of the literature; critical review of the manuscript.

Meng-Sha Ma: Collection, analysis, and interpretation of data; critical review of the manuscript.

Tao Chen: Approval of the final version of the manuscript; conception and planning of the study.

Li-Xin Fu: Critical review of the literature.

## Conflicts of interest

None declared.
